# Central Nervous System Toxoplasmosis is an Under‐Recognized Opportunistic infection in Uganda

**DOI:** 10.1155/jotm/2158978

**Published:** 2026-06-26

**Authors:** Nathan C. Bahr, John Kasibante, Laura Nsangi, Enock Kagimu, Kenneth Ssebambulidde, Morris K. Rutakingirwa, Lillian Tugume, Prashanth S. Ramachandran, Fiona Cresswell, David B. Meya, David R. Boulware, Michael R. Wilson, Jayne Ellis

**Affiliations:** ^1^ Division of Infectious Diseases & International Medicine, Department of Medicine, University of Minnesota, Minneapolis, Minnesota, USA, umn.edu; ^2^ Division of Infectious Diseases, Department of Medicine, University of Kansas, Kansas City, Kansas, USA, ku.edu; ^3^ Infectious Diseases Institute, College of Health Sciences, Makerere University, Kampala, Uganda, mak.ac.ug; ^4^ The Peter Doherty Institute for Infection and Immunity, University of Melbourne, Melbourne, Victoria, Australia, unimelb.edu.au; ^5^ Department of Neurology, The Royal Melbourne Hospital, Melbourne, Victoria, Australia, thermh.org.au; ^6^ Weill Institute for Neurosciences, Department of Neurology, University of California at San Francisco, San Francisco, California, USA, ucsf.edu; ^7^ Clinical Research Department, London School of Hygiene and Tropical Medicine, London, UK, lshtm.ac.uk

## Abstract

In Uganda*, Toxoplasma* meningoencephalitis remains underdiagnosed due to the low sensitivities and specificities of available diagnostics. In our recent publication, we identified 15 cases of possible *Toxoplasma gondii* meningoencephalitis by cerebrospinal fluid metagenomic next‐generation sequencing in patients with suspected meningitis. We herein discuss, in detail, these cases to highlight the ongoing limitations of utilizing clinical symptoms to diagnose *Toxoplasma gondii* meningoencephalitis, the importance of access to rapid diagnostics, and the frequency of toxoplasmosis as a possible co‐infection with other opportunistic diseases among people with advanced HIV.

## 1. Introduction

Individuals with advanced HIV disease (AHD), also known as acquired immunodeficiency syndrome (AIDS), are susceptible to *Toxoplasma gondii* (*T. gondii*) meningoencephalitis, which causes an estimated 15% of HIV‐associated in‐hospital deaths [1, 2]. The prevalence of *T. gondii* seropositivity is high in Sub‐Saharan Africa (∼45%), and in Uganda, more than 50% of people with HIV (PWH) are seropositive for this protozoan [3, 4]. PWH and latent toxoplasmosis infection are at significant risk of reactivation and subsequent development of symptomatic cerebral toxoplasmosis when CD4 T cell counts fall below 200 cells/mm^3^.

The diagnosis of cerebral toxoplasmosis in low‐resource settings relies mainly on a combination of clinical presentation, toxoplasma serology titers, and characteristic brain imaging findings in a susceptible host. This poses a challenge in most settings with high AHD burden because the clinical symptoms of *T. gondii* meningoencephalitis cannot conclusively differentiate it from meningoencephalitis caused by alternative opportunistic pathogens, including *Mycobacterium tuberculosis* and *Cryptococcus neoformans*. Furthermore, available serology and imaging tests have low specificity for active toxoplasmosis disease, and the rarely available commercial polymerase chain reaction (PCR) tests have variable sensitivities [5–7]. A study by Zawdzki *et al* showed that the sensitivity of computerized tomography (CT) scans to diagnose cerebral toxoplasmosis amongst PWH ranged between 65% and 82%, and specificity ranged from 75% to 82% [8]. Furthermore, the size and location of toxoplasmosis lesions significantly influence the likelihood that a radiologist will make a toxoplasmosis radiological diagnosis [8]. Serological immunoassay cutoffs to differentiate individuals with latent infection from active disease remain unclear, particularly in the context of AHD [6, 9]. In addition, these serological assays are influenced by the duration of infection and the *T. gondii* subtype [9]. In total, although diagnostic tools exist, none are perfect, and the typical laboratory‐based tests require the clinician to suspect CNS toxoplasmosis in order to request that the test be performed. Metagenomic next‐generation sequencing (mNGS) is pathogen agnostic and so has the ability to detect numerous pathogens which may cause meningoencephalitis; however, its application in diagnosing toxoplasmosis is scarcely reported [10, 11]. We previously identified fifteen diagnoses of cerebral toxoplasmosis cases using cerebrospinal fluid (CSF) mNGS amongst Ugandan adults with AHD and suspected tuberculous meningitis [10].

This short report highlights the impact of suboptimal toxoplasmosis diagnostics on outcomes amongst patients presenting with meningitis in a low‐income setting and the frequency of toxoplasmosis co‐infection with other opportunistic diseases in AHD.

## 2. Methods

Upon hospital admission, consecutive adults aged ≥ 18 years with suspected meningitis were consented and enrolled into a prospective observational cohort. All participants had a bedside test with a finger prick for cryptococcal antigen (CrAg) (IMMY, Norman, OK, USA) [12, 13], followed by a lumbar puncture to collect CSF for tests including the CrAg test, fungal culture, protein, glucose, lactate, white cell count, and Gram stain. Those who were CSF CrAg–negative also had bacterial culture, mycobacterial culture, TB lipoarabinomannan (LAM) testing (Alere, Waltham, Mass, USA), and GeneXpert MTB/Rif Ultra (Cepheid, Sunnyvale, CA, USA) performed. Urine Alere TB‐LAM and urine Xpert testing were also performed where possible [14]. Brain imaging and toxoplasma serologic testing were available and were utilized at the physician’s discretion; however, *T. gondii* PCR testing was not available. An additional 1 mL CSF was collected at the bedside in Zymo DNA/RNA Shield tubes, frozen at −80°C within 8 h of collection, and shipped in batches to the University of California, San Francisco, for mNGS. Metagenomic analysis was performed using an open source, cloud‐based metagenomics pipeline, CZID, which, after uploading raw data, removes host sequences and low‐quality, duplicate or low complexity reads in order to reduce contaminating sequences. Reads from bacterial organisms known to be found in low abundance in CSF mNGS datasets due to the environment, water, or specimen handing were normalized using compression ratios from the CZID pipeline to reduce contamination. For potential pathogens, abundance relative to the mean abundance of the entire cohort was also considered in a standardized way. Given the paucibacillary nature of TB in CSF, its abundance can fall below the reporting thresholds typically used in mNGS assay. Thus, we identified an optimal reporting threshold for TB using the training cohort and used this in the test cohort. The detailed mNGS workflow that was used to identify these cases has been previously described [15]. Positive *T. gondii* mNGS results were confirmed with *T. gondii* PCR testing. These results were not available in real‐time for clinical care.

Demographics, treatment, and outcomes were recorded. Participants were followed until hospital discharge and thereafter in an outpatient clinic. Data were presented as medians with interquartile ranges (IQRs) or proportions, with statistical testing performed with Fisher’s exact test for proportions. *p* values < 0.05 were considered significant. Data were analyzed using GraphPad Prism Version 10.4.1.

## 3. Results

From 2018 to 2019, 368 adults with suspected meningitis had DNA and RNA mNGS performed on their CSF. Overall, 4.1% (15/368) had *T. gondii* detected in CSF (95% CI: 2.3%–6.6%). Confirmatory PCR testing was positive for all 15 samples. Overall, 194 of 368 adults had data recorded on trimethoprim/sulphamethoxazole (TMP/SMX) prophylaxis, with 47% (91/194) of participants receiving TMP/SMX prophylaxis at admission. Only 20% (3/15) of the participants identified to have cerebral toxoplasmosis on mNGS were receiving TMP/SMX at admission. The incidence of cerebral toxoplasmosis was significantly higher among those not receiving TMP/SMX versus those prescribed TMP/SMX daily prophylaxis (11.7% [12/103] vs. 3.3% [3/91], *p* = 0.033, 95% CI). The TMP/SMX prophylaxis number needed to test (NNT) was 11.97 (95% CI: range: 1.15%–15.55%; odds ratio: 0.26, range: 0.01–0.85).

Demographics, CSF parameters, in‐hospital diagnosis, and outcomes for the 15 participants with cerebral toxoplasmosis are summarized in Table 1. The median age was 36 (IQR = 26–48) years, and 60% (9/15) were female. All had HIV. The majority (9/15) of patients were antiretroviral therapy (ART) naïve. Three were no longer on ART but had prior exposure, and three were on ART at admission. Individual participant details are summarized in Table 2.

**TABLE 1:** Summary statistics of patients with toxoplasmosis diagnosed using mNGS.

	Number *n* (%) (*N* = 15)	Median (IQR)
*Demographics*		
Age, years		36 (32.5, 41.8)
Female	9 (60%)	

*Antiretroviral therapy status*		
Naive	9 (60%)	
Discontinued	3 (20%)	
Taking ART at admission	3 (20%)	

*Trimethoprim (160 mg)/Sulfamethoxazole(800 mg) prophylaxis*		
On prophylaxis	3 (20%)	

*Clinical parameters*		
Fever	12 (80%)	
Weight loss	8 (53.3%)	
Headache	13 (86.7%)	
Seizures	4 (26.7%)	
Cough	6 (40%)	
Glasgow coma scale (< 15)^∗^	11 (73.3%)	
Focal neurological deficits	3 (20%)	

*CSF parameters*		
Opening pressure, cmH_2_O	13 (86.7%)	21 (12, 24)
Glucose, mg/dL	13 (86.7%)	60 (25, 100)
Lactate, mmol/L	7 (46.7%)	3.8 (3.15, 3.95)
Protein, mg/dL (normal range 15–60 mg/dL)	14 (93.3%)	108.55 (73.25, 134.9)

*CSF white blood cell count (cell/uL)*		
< 5 cells/μL	8 (53.3%)	
≥ 5 cell/μL	7 (46.7%)	70 (37.5, 117.5)

*CNS co-infections other than HIV*		
None	7 (46.7%)	
*Cryptococcus neoformans*	5 (33.3%)	
*Mycobacterium tuberculosis*	1 (6.7%)	
Herpes simplex virus‐2	1 (6.7%)	
Varicella‐zoster virus	1 (6.7%)	

*Mortality outcomes*		
Died	6/15 (40%)	
Days to death from admission		4 (4, 5)

*Note:* ART, antiretroviral therapy; CSF, cerebrospinal fluid; IQR, interquartile range.

Abbreviation: HIV, human immunodeficiency virus.

^∗^14 of 15 persons had GCS assessed at baseline.

**TABLE 2:** Cases with *Toxoplasma* detected by mNGS on CSF.

**Case**	**Demographics**	**Clinical presentation**		**CSF parameters.**						**Other diagnostics**	**Putative hospital diagnosis**	**Co-infections identified on mNGS**	**Follow-up and outcome**
		.	**GCS**	**Opening pressure (cmH**_**2**_ **0)**	**CrAg**	**White cells/μL**	**Protein (mg/dL)**	**Glucose (mg/dL)**	**Lactate (mmol/L)**				
1	34‐year‐old female, ART naïve	Oral thrush, fevers, seizures, altered mentation, hemiparesis, and meningism	14	8.5	Neg	< 5	23	31	2.1	Xpert negative	Bacterial meningitis	Herpes simplex virus‐2	Died in the second week of hospitalization. Postmortem blood HIV DNA PCR results were positive

2	36‐year‐old female, ART naïve	Fever, headache, and photophobia for 2 weeks	ND	21	Neg	< 5	12	ND	ND	Xpert negative	Bacterial meningitis	None	Died on Day 5 of treatment

3	27‐year‐old female, previously on ART	Fever, photophobia, headache, and visual loss for 4 days	14		Pos	145 (100% lymph)	29	25	ND	ND	Cryptococcal meningitis	*Cryptococcus neoformans*	Died on Day 8

4	30‐year‐old male on tenofovir/lamivudine/dolutegravir	Cryptococcal meningitis treatment 4 years earlier. Headaches for 3 weeks and vomiting for 1 week	ND	10	Pos	45 (10% lymph)	ND	low	2.7	N/A	Cryptococcal meningitis relapse	*Cryptococcus neoformans*	Discharged on Day 14 of care

5	47‐year‐old female, ART naïve	Headache, weight loss, fever, and vomiting	15	17	Pos	< 5	89	78	ND	Unremarkable chest X‐ray and abdominal U/S	Cryptococcal meningitis	*Cryptococcus neoformans*	Doing well 16 weeks from the baseline visit

6	42‐year‐old male, ART naïve	Fever and cough for 2 months, and headache for 1 month	14	20	?Pos	135 (100% lymph)	169	14	4	Chest x‐ray and abdominal ultrasound scan suggestive of tuberculosis	Possible TBM	*Cryptococcus neoformans*	Day 10 CSF culture showed 34,340 *Cryptococcus neoformans* colony‐forming units/mL. Lost to follow‐up

7	35‐year‐old male on tenofovir/lamivudine/efavirenz	Headache, fever, night sweats, and weight loss for 3 weeks. Photophobia and vomiting for 4 days	15	35	Neg	40	174	41	3.9	Negative Xpert ultra and Alere TB‐LAM tests	Possible TBM	*Cryptococcus neoformans*	Doing well 24 weeks from the baseline visit

8	40‐year‐old male, previously on ART	Weight loss for 3 weeks. Confusion and cough for 2 weeks. Fever and night sweats for 1 week. Vomiting for 2 days	15	27	Neg	35	71	100	3.6	Xpert negative	Possible TBM	None	Hospitalized through Day 14

9	48‐year‐old female, previously on ART	Weight loss and cough for 3 months. Headache and confusion for 5 days	10	25	Neg	< 5	94.8	148	3.8	N/A	Possible TBM	None	Discharged on Day 8

10	47‐year‐old female, ART naïve	Weight loss for 1 month. Headache, fever, cough, and night sweats for 2 weeks. Confusion for 4 days	11	12	Neg	25	136	73	ND	N/A	Possible TBM	None	Left the hospital on Day 6 of care

11	41‐year‐old female, ART naïve	Cough for two months. Headache and fever for 1 month. Seizures and confusion for 1 week. Had lymphadenitis, nuchal rigidity, focal neurological deficits, and cranial nerve palsy	11	23	Neg	100 (93% lymph and 7% neut)	122.3	6.7	ND	CSF and urine Xpert and TB‐LAM negative. Chest X‐ray and abdominal ultrasound scan suggestive of focal TB lesions	Possible TBM	Varicella‐Zoster virus	She died on Day 4

12	32‐year‐old male previously on ART	Headache, confusion, fever, night sweats, weight loss, and cough	10	ND	Neg	< 5	80	25.2	ND	N/A	Possible TBM	None	Left the hospital against medical advice on Day 2

13	31‐year‐old female, ART naïve	Headache, visual changes, confusion, fever, night sweats, seizures, and vomiting	8	12	Neg	< 5	154	60	4.4	N/A	Possible TBM	None	Died on Day 3

14	26‐year‐old male, on tenofovir/lamivudine/dolutegravir	Weight loss for 2 months. Cough and fever for 1 month. Headache and night sweats for 3 weeks. Confusion for 2 weeks. Vomiting for 1 week. Seizure for 2 days. Had nuchal rigidity, focal neurological deficits, anisocoria, generalized lymphadenopathy, and reduced lung air entry with crepitation bilaterally	10	24	Neg	< 5	123.1	102.6	ND	CSF Gene Xpert ultra negative. Brain CT scan unremarkable. Chest X‐ray suggestive of TB	Probable TBM	None	Died on Day 4

15	36‐year‐old female, ART naïve	Weight loss and night sweats for 1 month. Headache for 2 weeks. Confusion for 4 days. Had focal neurological deficits, left cranial nerve seven palsy, nuchal rigidity, and meningism	14	23	Neg	< 5	131.6	ND	ND	CSF and urine Xpert ultra plus urine Alere TB‐LAM were positive	Definitive TBM	*Mycobacteria tuberculosis*	Fully recovered at 24 weeks postinitial diagnosis

*Note:* CSF culture and gram stain results are indicated where relevant. All patients received fixed‐dose combination tablets of trimethoprim 160 mg/sulfamethoxazole 800 mg as toxoplasmosis prophylaxis on hospitalization. ?pos, indeterminate result; ART, antiretroviral therapy; CrAg, cryptococcal antigen; CSF, cerebrospinal fluid; DNA, deoxyribonucleic acid; CT scan, computed tomography scan; LAM lipoarabinomannan; Neg, negative; Pos, positive; RNA, ribonucleic acid; TB, tuberculosis; TBM, tuberculous meningitis.

Abbreviations: GCS, Glasgow coma scale; HIV, human immunodeficiency virus; mNGS, metagenomic next‐generation sequencing; N/A, not applicable; ND, not done; ZN, Ziehl–Nielsen stain.

The most common presenting symptoms were headache (87%), fever (80%), weight loss (53%), cough (40%), and seizures (27%). Overall, 11 of 14 participants (79%) had a Glasgow coma scale score of < 15, and only 20% (3/15) had focal neurological deficits. Symptom duration before admission varied from 4 days to 4 months.

In‐hospital diagnoses made by the attending clinicians included TB meningitis (*n* = 10, 1/10 was microbiologically confirmed TB meningitis), confirmed cryptococcal meningitis (*n* = 3), symptomatic cryptococcal antigenemia (*n* = 2), and presumed bacterial meningitis (*n* = 2). None of the patients had brain imaging, toxoplasma serology, real‐time CSF *T. gondii* PCR testing, or was treated for cerebral toxoplasmosis.

Co‐infection with multiple pathogens occurred in 8 of 15 persons where mNGS detected *T. gondii* nucleic acid alongside another significant opportunistic pathogen. Five had *C. neoformans* co‐infection, and one participant each had co‐infection with *Mycobacterium tuberculosis* (*n* = 1), Herpes simplex virus‐2 (*n* = 1), or varicella‐zoster virus (*n* = 1). Of the five participants who had *T. gondii co-infection* with cryptococcosis evident by cryptococcal antigenemia in blood, three were CSF CrAg‐positive, and two of these were CSF culture positive. In seven patients, mNGS detected *T. gondii* as a single pathogen, of whom three died within 5 days of admission.

Prophylactic TMP/SMX at hospitalization was initiated once daily for all patients. Outcomes were poor, with six participants dying during hospitalization at a median time to death of 4 days (IQR: 4‐5 days) from hospital admission. Three patients withdrew from care, and three patients (20%) continuing prophylactic TMP/SMX were alive 16 weeks after admission without appropriate cerebral toxoplasmosis treatment.

## 4. Discussion

We previously reported that we detected *T. gondii* in 15 CSF samples by mNGS, looking broadly for neuroinfections in a large cohort of adults with HIV presenting with suspected TB meningitis [15]. Herein, we provide a more detailed assessment of the clinical trajectories of these 15 individuals to better understand the diagnostic challenges of cerebral toxoplasmosis in a low‐resource setting and the impact on treatment outcomes. The majority of cases where *T. gondii* was detected were co‐infections with *T. gondii* detected alongside another significant opportunistic pathogen. *Cryptococcus* was the most frequent co‐pathogen, highlighting the ongoing importance of co‐infections amongst adults presenting with AHD and suspected meningitis [16, 17]. Whether *T. gondii* is an important pathogen in these cases is unclear, but the lack of response to typical therapy could indicate co‐infection, even amongst diseases with significant baseline mortality such as cryptococcal meningitis and TB meningitis.

The *T. gondii* detection prevalence of 4.1% we report in this study is comparable to the 3% *T. gondii* CSF PCR positivity our research team reported in 2015 in a cohort of hospitalized adults presenting with suspected meningitis [17]. The comparative prevalence rates indicate a stable level of possible CNS toxoplasmosis and the ongoing need to improve preventative therapy to eliminate *T. gondii*‐related mortality and morbidity. The TMP/SMX prophylaxis NNT of twelve persons in this study further emphasizes the importance of *T. gondii* as an opportunistic infection among people with AHD in Uganda.

Uganda has achieved considerable progress in meeting the United Nations Program on HIV/AIDS (UNAIDS) “90‐90‐90” targets, and yet, presentations to the hospital with HIV‐associated meningitis remain common, with our team performing lumbar punctures for 368 adults with suspected meningitis from 2018 to 2019. Only 30% of our cohort were taking ART at hospitalization; this proportion is comparable with other studies amongst those presenting with meningitis symptoms [16]. These data highlight the ongoing challenges of late HIV presentation and diagnosis and suboptimal retention in HIV care, which underpin the persistent burden of AHD.

None of the fifteen cases of cerebral toxoplasmosis was diagnosed during hospitalization. There is a notable clinical overlap in symptoms, signs, and CSF abnormalities between cerebral toxoplasmosis, bacterial, cryptococcal, and tuberculous meningitis [15, 17]. Indeed > 80% of participants with *Toxoplasma* detected in CSF by mNGS in this study reported headache and fever largely indistinguishable from other causes of infectious meningitis. It is also noteworthy that a minority (3/15, 20%) of cases with *T. gondii* in their CSF had focal neurological symptoms, which one assumes is the hallmark feature of cerebral toxoplasmosis but can also be present in other CNS infections.

Although the number of cases presented is not large, nor systematically defined, our findings should reiterate the need for clinicians to have a low threshold to treat toxoplasmosis, including in patients failing to respond to treatment for cryptococcal, TB, or bacterial meningitis. In our setting, *C. neoformans* and *M. tuberculosis* remain the most common causes of HIV‐associated meningoencephalitis [17]. However, it seems likely that CNS toxoplasmosis co‐infection is going undiagnosed and untreated in at least some adults presenting with HIV‐associated meningitis.

Conversely, our participants with mNGS confirmed cerebral toxoplasmosis were more likely to be ART naïve, to report seizures at baseline, have a GCS < 15, and have normal CSF parameters when compared to our previous cohorts with all‐cause suspected meningitis [13]. These clinical characteristics may therefore trigger clinical suspicion of possible *T. gondii* infections, even in the presence of another confirmed opportunistic pathogen. We previously showed that PCR used as a confirmatory test for *T. gondii* detected by mNGS had a 100% concordance [15]. However, given the limited sensitivity that PCR (and by extension, other molecular detection tests such as mNGS) have for detecting *T. gondii* in CSF (i.e., 50%–86% sensitive), it is likely that our cohort had additional cases that might have been missed by mNGS and may have instead been detected if neuroimaging and PCR testing were more widely available [5, 11, 18]. Here is a clear need for improved and more accessible point‐of‐care diagnostic tests for toxoplasmosis to reduce reliance on clinical parameters for diagnosis.

None of the reported patients received appropriate cerebral toxoplasmosis treatment, which may have contributed to early mortality and further highlights the need for early toxoplasmosis diagnosis and treatment. Notably, TMP/SMX treatment leads to radiographic improvement in 80%–90% of cases, further emphasizing that good outcomes are possible with early treatment [19]. Three patients (Cases 5, 7, and 15) in whom *T. gondii* was detected in CSF by mNGS were not treated but were alive beyond 16 weeks of initial presentation. This is in contrast to earlier descriptions of untreated cerebral toxoplasmosis in the early ART era, which was considered to be uniformly fatal [19]; recovery amongst our participants could be attributed to rapid and effective immune recovery following integrase‐based ART initiation alongside a 960 mg prophylactic dose of TMP/SMX. It may be, however, that in these cases, cryptococcal meningitis or TB meningitis being appropriately treated was of more consequence than the lack of treatment for *T. gondii*. Detection of *T. gondii* by mNGS (or PCR) alone may not necessarily reflect disease.

Molecular detection of *T. gondii* must be considered in the overall clinical context. For instance, in a person with cryptococcal meningitis, improving rapidly on standard therapy, *T. gondii* in CSF may not be meaningful. However, in a person with cryptococcal meningitis doing poorly on therapy with radiographic signs typical of CNS toxoplasmosis (e.g., focal, contrast‐enhancing mass lesions) in whom focal neurological findings on examination made the pretest probability of CNS toxoplasmosis high, the positive *T. gondii* molecular test is likely to be clinically important. Considering this approach in the context of the three patients in this study who improved without treatment for toxoplasmosis, the *T. gondii* result may be less clinically important.

Our study limitations include a retrospective design and some missing data, including CD4 cell counts, as well as an overall small number of cases in which *T. gondii* was detected. While standardized brain imaging was not performed, only three patients had focal neurologic deficits on examination, which would have prompted brain imaging in this setting.

In conclusion, cerebral toxoplasmosis remains an important opportunistic infection in the HIV “test and treat” era, especially among patients who are ART naïve. Not all patients with cerebral toxoplasmosis present with CNS localizing signs. Therefore, high clinical suspicion to increase *T*. *gondii* testing with serology, imaging, and molecular tests may improve diagnosis, rapid initiation of appropriate therapy, and outcomes. Broader access to brain imaging and PCR is particularly important. Furthermore, mNGS may have diagnostic potential, but further study is needed to understand diagnostic accuracy, cost‐effectiveness, and usability in our setting. mNGS cost and infrastructure requirements must improve for mNGS to become a broadly accessible option. In the absence of definitive diagnostic testing, empiric therapeutic dosing of TMP/SMX in high‐risk hospitalized adults presenting with AHD and suspected meningitis, with no prior prophylactic antimicrobial therapy, could be a reasonable strategy and warrants further investigation.

## Funding

The authors report funding from the National Institutes of Health, the National Institute of Neurological Diseases and Stroke (Grant nos. R01NS086312 and K23NS110470), the National Institute of Allergy and Infectious Diseases (Grant no. R01AI145437), and the Westridge Foundation (MRW). FVC is supported by Wellcome (Grant no. 300088/Z/23/Z), and JE is supported by Wellcome Trust Clinical PhD Fellowship (Grant no. 203905/Z/16/Z).

## Conflicts of Interest

MRW is a co‐founder and board member of Delve Bio. The remaining authors declare no conflicts of interest.

## Data Availability

The data that support the findings of this study are available from the corresponding author upon reasonable request.
